# Plasma 25-hydroxyvitamin D_2_ and D_3_ levels and incidence of postoperative atrial fibrillation

**DOI:** 10.1017/jns.2015.38

**Published:** 2016-02-15

**Authors:** G. V. Skuladottir, A. Cohen, D. O. Arnar, D. M. Hougaard, B. Torfason, R. Palsson, O. S. Indridason

**Affiliations:** 1 Department of Physiology, School of Health Sciences, University of Iceland, Reykjavik, Iceland; 2 Faculty of Medicine, School of Health Sciences, University of Iceland, Reykjavik, Iceland; 3 Department of Congenital Disorders, Statens Serum Institute, Copenhagen, Denmark; 4 Internal Medicine Services, Landspitali – The National University Hospital of Iceland, Reykjavik, Iceland; 5 Surgical Services, Landspitali – The National University Hospital of Iceland, Reykjavik, Iceland

**Keywords:** Atrial fibrillation, Heart surgery, Postoperative state, Vitamin D, 1,25(OH)_2_D, 1,25-dihydroxyvitamin D, 25(OH)D, 25-hydroxyvitamin D, AF, atrial fibrillation, POAF, postoperative atrial fibrillation, SR, sinus rhythm

## Abstract

Low circulating levels of total 25-hydroxyvitamin D (25(OH)D) have been associated with an increased risk of adverse effects after cardiac surgery. The metabolites, 25(OH)D_2_ and 25(OH)D_3_, provide a good index of vitamin D status. In this study, we examined the association between preoperative plasma levels of total 25(OH)D, 25(OH)D_2_ and 25(OH)D_3_ and the risk of postoperative atrial fibrillation (POAF) following open heart surgery. The levels of plasma 25(OH)D_2_ and 25(OH)D_3_ in 118 patients, who underwent coronary artery bypass grafting and/or valvular surgery, were measured immediately prior to surgery and on postoperative day 3 by liquid chromatography–tandem mass spectrometry. Patients who developed POAF had higher median plasma levels of 25(OH)D_2_ than those who remained in sinus rhythm (SR) (*P* = 0·003), but no significant difference was noted in levels of 25(OH)D_3_ or total 25(OH)D between the two groups (*P* > 0·05). By univariate analysis, patients with total 25(OH)D and 25(OH)D_2_ levels above the median had higher frequency of POAF (*P* < 0·05) and the incidence of POAF increased significantly with each higher quartile of preoperative plasma levels of 25(OH)D_2_ (*P* = 0·001), an association that was independent of confounding factors. In both the SR and POAF groups, the median plasma levels of 25(OH)D_2_, 25(OH)D_3_ and total 25(OH)D were lower (*P* < 0·05) on the third postoperative day compared with preoperatively. Our findings demonstrate that higher plasma levels of 25(OH)D_2_ are associated with increased risk of POAF, while this is not the case for 25(OH)D_3_ or total 25(OH)D. The reason for these discrepant results is not clear but warrants further study.

Low levels of total 25-hydroxyvitamin D (25(OH)D), the major circulating form of vitamin D, have been associated with various adverse outcomes following open heart surgery, including in-hospital death, myocardial infarction, low cardiac output states and stroke^(^^1^^–^^3^^)^.

The most important vitamin D compounds are vitamin D_2_ (ergocalciferol), found in plants and consumed as a supplement or in fortified foods, and vitamin D_3_ (cholecalciferol), which is synthesised in the human epidermis under the influence of sunlight or consumed in oily fish, fortified foods or as a supplement. 25-Hydroxyergocalciferol (25(OH)D_2_) and 25-hydroxycholecalciferol (25(OH)D_3_), which are generally considered to reflect an individual's overall vitamin D status, are produced by hydroxylation in the liver^(^^4^^)^. In the kidney, part of total 25(OH)D is converted to the active form of vitamin D, 1,25-dihydroxyvitamin D (1,25(OH)_2_D), which plays an important role in the regulation of mineral homeostasis and has beneficial effects on cardiovascular function and the immune system^(^^5^^)^.

A relationship between vitamin D deficiency and the development of atrial fibrillation (AF) has been suggested, although reported studies have yielded somewhat conflicting results^(^^6^^–^^8^^)^. However, no studies exist on the possible role of vitamin D metabolites in the development of postoperative AF (POAF), which is surprising in view of the association of vitamin D deficiency with adverse outcomes after cardiac surgery^(^^1^^–^^3^^)^.

Therefore, this study was carried out to investigate whether higher preoperative plasma levels of 25(OH)D_2_, 25(OH)D_3_ and total 25(OH)D are associated with lower incidence of POAF in patients undergoing open heart surgery.

## Materials and methods

### Subjects

This study was based on prospectively collected data from a randomised, double-blind, placebo-controlled clinical trial designed to examine the effect of *n*-3 long-chain PUFA on the occurrence of POAF in patients who underwent coronary artery bypass grafting and/or valvular repair surgery. The study was approved by the Bioethics Committee of Landspitali – The National University Hospital of Iceland (62/2004). All patients provided written informed consent. Details of the study design have been published previously^(^^9^^)^. In brief, patients scheduled for elective or semi-urgent open heart surgery were evaluated for participation. Patients younger than 40 years of age, those with a history of supraventricular arrhythmia or use of the antiarrhythmic medications amiodarone and/or sotalol, and patients undergoing emergency surgery were excluded. Prior to surgery, all participants answered a questionnaire on lifestyle issues, including consumption of fish and cod liver oil, intake of supplemental *n*-3 long-chain PUFA capsules, smoking habits, alcohol consumption, height, body weight and medication use. The active treatment consisted of two Omega-3 Forte capsules twice daily, providing a total of 1240 mg of EPA ethyl ester and 1000 mg of DHA ethyl ester but no vitamin D (Omega Forte; Lysi Inc.). The placebo group received 2 g of olive oil in identical capsules (Lysi Inc.). The treatment was initiated 5–7 d before the scheduled date of surgery and continued until the day of discharge from the hospital or for a maximum duration of 2 weeks after the surgery. All patients underwent continuous electrocardiaographic monitoring while hospitalised and the study endpoint, POAF, was defined as an episode of AF lasting more than 5 min.

### Measurement of plasma 25-hydroxyvitamin D_2_ and D_3_

Plasma samples obtained both immediately before the surgery (preoperatively) and on the third postoperative day (postoperatively), when the risk for POAF is high, were available for 118 of the 168 original randomised controlled trial participants. The plasma samples were stored at −76°C until analysis for 25(OH)D_2_ and 25(OH)D_3_ using an MS/MS Vitamin D Kit (Perkin Elmer), and carried out as described previously^(^^10^^)^. Briefly, 30 µl of serum were deproteinised in microtitre plates using 120 µl acetonitrile containing [^2^H_3_]25(OH)D_2_ and [^2^H_3_]25(OH)D_3_ as internal standards. The supernatants were transferred to fresh plates and dried under a gentle flow of N_2_. Subsequently, the samples were derivatised using 4-phenyl-1,2,4-triazoline-3,5-dione (PTAD) dissolved in acetonitrile. The derivatisation reaction was quenched in quenching solution and the samples were subjected to liquid chromatography–tandem mass spectrometry (LC-MS/MS) analysis. The LC-MS/MS system consisted of a CTC PAL Autosampler (CTC Analytics), a Thermo Surveyor LC pump and a Thermo TSQ Ultra Triple Quadrupole mass spectrometer (Thermo Scientific). Separation was achieved using a Thermo Gold Hypersil C18 column (50 × 2·1 mm, 3 µm). The analytes were detected as methylamine adducts and the following transitions were used: 619·3/298·1 and 607·3/298·1 for 25(OH)D_2_ and 25(OH)D_3_, respectively, 622·3/301·1 and 610·3/298·1 for 25(OH)D_2_ and 25(OH)D_3_ internal standards, respectively, and 625·3/298·1 and 613·3/298·1 for 25(OH)D_2_ and 25(OH)D_3_ calibration standards, respectively. The assay does not differentiate between epi-25(OH)D_3_ and 25(OH)D_3_. Thus the level reported for 25(OH)D_3_ includes any epi form if present, but the detected level of 25(OH)D_2_ is unaffected by the epi form. Total plasma 25(OH)D was calculated as the sum of the plasma levels of 25(OH)D_2_ and 25(OH)D_3_. The correctness of the method was confirmed through participation in the external control program DEQAS.

### Measurement of plasma postoperative C-reactive protein

Routine postoperative care included daily measurements of C-reactive protein using a sandwich enzyme immunoassay (Vitros 5.1 FS Chemistry System; Ortho-Clinical Diagnostic).

### Statistical analyses

Mann–Whitney or χ^2^ tests were used to compare the sinus rhythm (SR) and POAF groups with respect to baseline characteristics. The Wilcoxon signed-rank test was used to compare the differences in plasma levels of 25(OH)D_2_, 25(OH)D_3_ and total 25(OH)D between time points in the two study groups. To examine the association between POAF and the preoperative plasma levels of 25(OH)D_2_, 25(OH)D_3_ and total 25(OH)D, we compared the rate of POAF between dichotomised levels and quartiles of plasma 25(OH)D_2_, 25(OH)D_3_ and total 25(OH)D using the χ^2^ test and Somers’ *d* test for ordinal variables. A stepwise multivariable logistic regression was used to examine factors associated with POAF. In the original investigation, there was no difference in POAF incidence between the active treatment and placebo groups, and no association between treatment and outcome. Hence, we have ignored treatment allocation in our analyses. Multivariable linear regression analysis was used to assess predictors of the preoperative plasma levels of 25(OH)D_2_ and 25(OH)D_3_ in the whole study group. Data are presented as medians and ranges, percentages or means with their standard errors. A two-sided *P* value <0·05 was considered statistically significant. All statistical analyses were carried out using SPSS (version 21.0; IBM Corporation).

## Results

### Baseline characteristics

The baseline characteristics of the patients in the SR (*n* 52) and POAF (*n* 66) groups are shown in Table 1. The patients with POAF were older (*P* = 0·001), they comprised fewer smokers (*P* = 0·041), and they consumed fish more frequently (*P* = 0·034) than the SR group. Immediately before the surgery and on the third postoperative day, the median plasma level of 25(OH)D_2_ was higher in the POAF group than in the SR group (*P* = 0·003 and *P* = 0·040, respectively) (Table 2). However, the differences in levels of 25(OH)D_3_ or total 25(OH)D between the two groups were not significant. In both the SR and POAF groups, the median plasma levels of 25(OH)D_2_, 25(OH)D_3_ and total 25(OH)D were lower (*P* < 0·05) on the third postoperative day compared with the preoperative values (Table 2).

**Table 1.** Characteristics of the study subjects (Medians and ranges, or percentages)

	SR group (*n* 52)		POAF group (*n* 66)		
	Median	Range	Median	Range	P*
Age (years)	64·0	43–79	70·5	45–82	0·001
BMI (kg/m^2^)	28·0	20·9–41·3	27·1	21·0–38·1	0·86
Sex (% men)	78·8		81·8		0·69
Smokers (%)	26·9		12·1		0·04
Alcohol consumption ≥once per week (%)	67·3		74·2		0·42
Fish intake ≥once a week (%)	59·6		78·8		0·03
Use of liquid cod liver oil (%)†	50·0		54·5		0·70
Use of *n*-3 long-chain PUFA capsules (%)†	19·2		31·8		0·14
*n*-3 Long-chain PUFA treatment (%)	50·0		53·0		0·74
Diabetes (%)	17·3		12·1		0·43
Treatment with statins (%)	84·6		72·7		0·12
CABG only (%)	78·8		66·7		0·15
Total blood volume in drains (ml)	710	175–3070	800	96–4980	0·58

SR, sinus rhythm; POAF, postoperative atrial fibrillation; CABG, coronary artery bypass grafting.

* Compared with the SR group (Mann–Whitney test or χ^2^ test).

† Prior to study treatment.

**Table 2.** Plasma vitamin D levels of patients in the sinus rhythm (SR) and postoperative atrial fibrillation (POAF) groups, immediately before cardiac surgery (preoperative) and on the third postoperative day (postoperative) (Medians and ranges)

	Preoperative		Postoperative		
	Median	Range	Median	Range	P*
SR group (*n* 52)					
25(OH)D_2_ (nmol/l)	0·8	0·0–4·4	0·75	0·0–3·2	0·003
25(OH)D_3_ (nmol/l)	37·8	7·4–89·1	29·4	7·6–71·3	<0·001
Total 25(OH)D (nmol/l)	39·3	8·1–90·5	30·5	8·6–72·2	<0·001
POAF group (*n* 66)					
25(OH)D_2_ (nmol/l)	1·3†	0·0–20·8	1·0†	0·0–13·2	<0·001
25(OH)D_3_ (nmol/l)	51·6	8·6–83·5	37·7	7·3–76·0	<0·001
Total 25(OH)D (nmol/l)	52·4	8·6–84·9	38·3	7·3–77·3	<0·001

25(OH)D, 25-hydroxyvitamin D.

* Compared with preoperative 25(OH)D_2_, 25(OH)D_3_ or total 25(OH)D levels (Wilcoxon signed-rank test).

† Median value was significantly different from that of the SR group (*P* < 0·05; Mann–Whitney test).

### Association of plasma 25-hydroxyvitamin D_2_ and D_3_ levels with postoperative atrial fibrillation

**Fig. 1.**
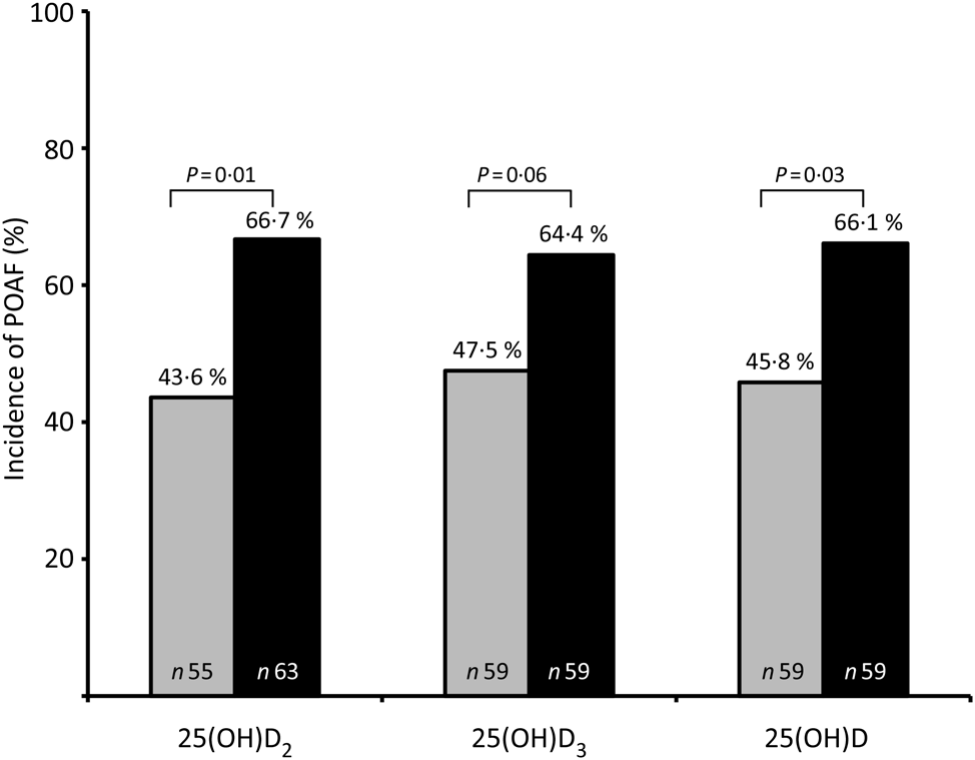
Incidence of postoperative atrial fibrillation (POAF) according to dichotomised preoperative plasma levels of 25-hydroxyvitamin D_2_ (25(OH)D_2_), 25(OH)D_3_, and total 25(OH)D in patients undergoing open heart surgery. Median levels ≥1·1 nmol/l for 25(OH)D_2_; ≥45·9 nmol/l for 25(OH)D_3_; ≥47·1 nmol/l for total 25(OH)D. Data were analysed by Pearson's χ^2^. 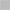, Below median; ■, above median.

**Fig. 2.**
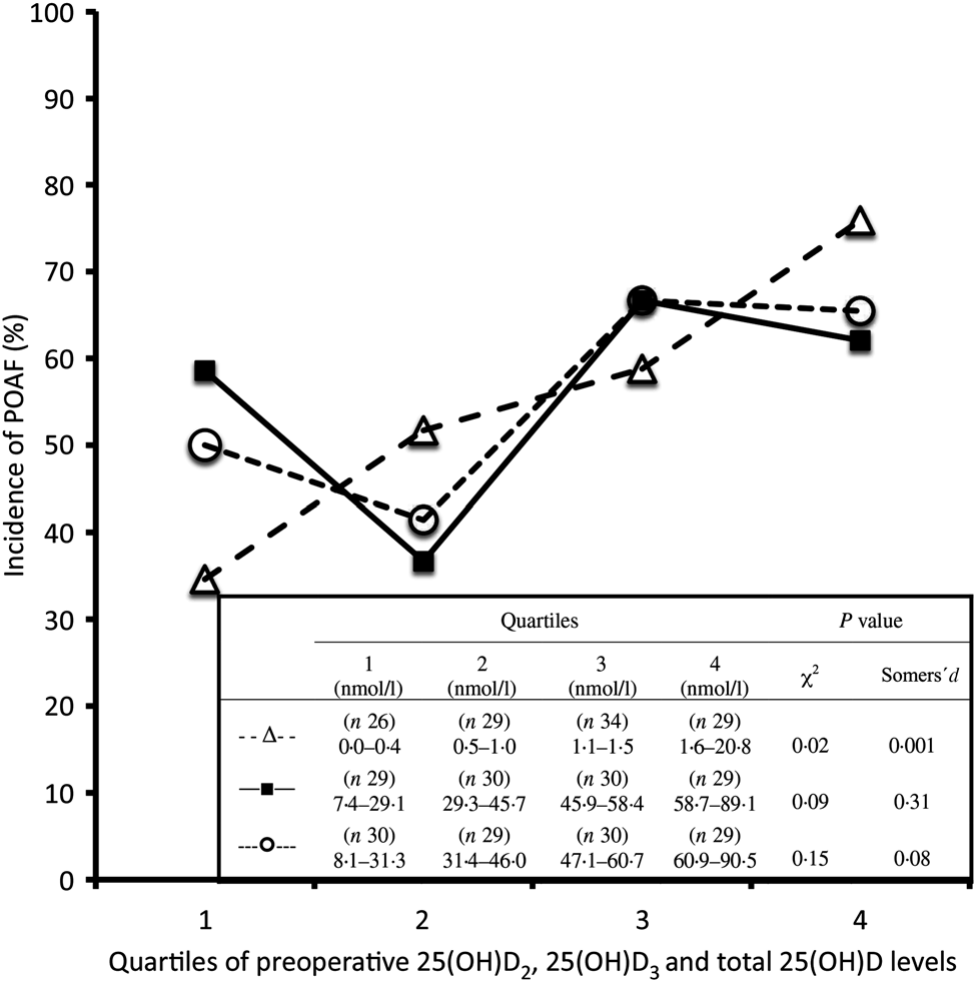
Incidence of postoperative atrial fibrillation (POAF) according to quartiles of the preoperative plasma levels (nmol/l) of 25-hydroxyvitamin D_2_ (25(OH)D_2_; --Δ--), 25(OH)D_3_ (–■–) and total 25(OH)D (--○--) in patients undergoing open heart surgery. Data were analysed by Pearson's χ^2^ and Somers’ *d* statistics for trend in association between ordinal variables.

The incidence of POAF was higher (*P* < 0·05) in those with preoperative plasma levels above the median for 25(OH)D_2_ (≥1·1 nmol/l) and total 25(OH)D (≥47·1 nmol/l) compared with those below the median, whereas the difference was of borderline significance (*P* = 0·06) for 25(OH)D_3_ (≥45·9 nmol/l) (Fig. 1). When the patients were divided into quartiles based on preoperative plasma levels of 25(OH)D_2_ and 25(OH)D_3_, the POAF incidence differed for the 25(OH)D_2_ levels (*P* = 0·020), with a significant linear trend for an increasing incidence of POAF observed with the higher quartiles (*P* = 0·001) (Fig. 2). The difference in POAF incidence observed between the quartiles of 25(OH)D_3_ and total 25(OH)D levels demonstrated a non-significant J-shaped curve (*P* > 0·05).

Multivariable logistic regression analysis, adjusting for factors known or presumed to affect POAF, showed that higher preoperative plasma levels of 25(OH)D_2_ were associated with higher risk of developing POAF with OR of 2·747 (95 % CI 1·121, 6·734; *P* = 0·027), while no significant association between POAF and plasma levels of 25(OH)D_3_ or total 25(OH)D was observed (Table 3).

**Table 3.** Multivariable logistic regression analysis of factors associated with postoperative atrial fibrillation*

	*R*^2^	OR	95 % CI	*P*
Age (years)	0·127	1·07	1·03, 1·12	0·001
Peak postoperative CRP (mg/l)	0·195	1·01	1·00, 1·01	0·02
Plasma preoperative DHA (%)	0·241	2·63	1·10, 6·28	0·03
Smoking (reference, non-smoker)	0·275	0·37	0·13, 1·05	0·06
Type of surgery (reference, valvular or complex procedure)	0·304	0·42	0·16, 1·12	0·08
BMI (kg/m^2^)	0·308	0·96	0·85, 1·08	0·47
25(OH)D_2_ (reference, lower half)†	0·352	2·75	1·12, 6·73	0·03
25(OH)D_3_ (reference, lower half)†	0·309	1·10	0·45, 2·66	0·83
Total 25(OH)D (reference, lower half)†	0·311	1·31	0·54, 3·16	0·55

*R*^2^, Nagelkerke's *R*^2^; CRP, C-reactive protein; 25(OH)D, 25-hydroxyvitamin D.

* Stepwise forward selection of variables, 25(OH)D_2_, 25(OH)D_3_ and total 25(OH)D added in the final step in three different models. Cumulative *R*^2^ for each step of the regression analysis.

† Preoperative plasma 25(OH)D_2_, 25(OH)D_3_ and total 25(OH)D levels dichotomised at the median.

### Factors predicting the plasma levels of 25-hydroxyvitamin D_2_ and D_3_

In the multivariable linear regression model, the preoperative plasma level of 25(OH)D_2_ was negatively associated with alcohol consumption (β = −0·210; *P* = 0·024), but no association with the *n*-3 long-chain PUFA treatment and other factors was detected. To the contrary, 25(OH)D_3_ was positively associated with cod liver oil supplementation (β = 0·406; *P* < 0·001) and alcohol consumption (β = 0·193; *P* = 0·022), but negatively associated with diabetes (β = −0·217; *P* = 0·012) and smoking (β = −0·206; *P* = 0·014).

## Discussion

In this study, we found higher preoperative plasma 25(OH)D_2_ levels to be associated with an increased risk of POAF. In contrast, we did not observe an association between plasma levels of 25(OH)D_3_ or total 25(OH)D and the incidence of POAF. The plasma levels of both 25(OH)D_2_ and 25(OH)D_3_ decreased in the early postoperative period.

Previous studies suggest that deficiency of total 25(OH)D is associated with increased prevalence of electrocardiographic abnormalities^(^^11^^)^. In a recent prospective cohort study of patients undergoing cardiac surgery, low total 25(OH)D levels were independently associated with the risk of major cardiac and cerebrovascular events (MACCE)^(^^1^^)^, although the investigators did not report whether a relationship existed between 25(OH)D and the development of POAF. However, previous work has elucidated a potential role of total 25(OH)D in the development of non-valvular AF^(^^6^^,^^7^^)^. The results of the present study, following adjustment for age, BMI, smoking, peak postoperative C-reactive protein, preoperative plasma DHA level and valvular surgery or complex surgical procedure^(^^12^^)^, showed that higher preoperative plasma levels of 25(OH)D_2_ were related to the development of POAF. To the contrary, neither 25(OH)D_3_ nor total 25(OH)D levels were associated with POAF. This finding was somewhat surprising, since median plasma 25(OH)D_2_ levels in humans are generally low, as exemplified by the patients in the present study. In the POAF group, the highest plasma 25(OH)D_2_ level was 20·8 nmol/l compared with 4·4 nmol/l in the SR group. A recent study demonstrated the distribution of serum 25(OH)D_2_ concentrations in samples of Irish adults (*n* 884), of whom 78·8 % had concentrations above the limit of quantification (1·43 nmol/l), the median concentration being 2·96 nmol/l, and the maximum concentration 27·64 nmol/l^(^^13^^)^. According to the authors, the distribution of the serum 25(OH)D_2_ data suggests that vitamin D_2_ is ubiquitous in the diet, regardless of food sources. It is therefore likely that dietary differences between study populations explain the observed difference in the levels of 25(OH)D_2_. While a causal link for the observed association might be sought in the effects of 25(OH)D_2_ on cardiac function or rhythm, oxidative stress or inflammatory pathways, this remains speculative and it is important that our findings be confirmed in larger studies.

Our study did not have enough power to examine MACCE in relation to vitamin D levels. Prior studies evaluating this association have not examined 25(OH)D_2_ and 25(OH)D_3_ separately, only total 25(OH)D^(^^6^^,^^7^^)^. Hence, it remains unclear whether the effect of the two forms of vitamin D might also be different with respect to MACCE. Nevertheless, it is possible that failure to account for a differential effect of 25(OH)D_2_ and 25(OH)D_3_ may explain inconsistency in the results of studies examining the association between vitamin D and AF, an important issue that needs to be addressed in future studies.

Among the conditions that have been linked to deficiency of circulating total 25(OH)D are type 2 diabetes and hypertension, although randomised clinical trials and meta-analyses have yielded inconclusive results^(^^14^^,^^15^^)^. Recent studies have also shown that exposure to cigarette smoke is associated with lower circulating levels of total 25(OH)D and 25(OH)D_3_^(^^16^^,^^17^^)^. Our findings are consistent with data suggesting that low 25(OH)D_3_ levels are associated with diabetes and cigarette smoking^(^^14^^,^^17^^)^. It has long been known that excessive alcohol consumption results in perturbations in vitamin D metabolism and that individuals suffering from chronic alcoholism usually have lower levels of circulating total 25(OH)D than non-drinkers^(^^18^^)^. However, the results of our study suggest that moderate alcohol consumption may result in lower levels of 25(OH)D_2_ and higher 25(OH)D_3_ levels, the latter of which is in line with previous reports^(^^19^^,^^20^^)^. Thus, our findings indicate that both lifestyle and disease states are related to circulating levels of vitamin D metabolites. Decrease in the plasma 25(OH)D_3_ level following cardiac surgery may reflect increased conversion into the active form, 1,25(OH)_2_D^(^^1^^,^^20^^)^. The effects of anaesthesia and other factors associated with open heart surgery, such as fluid administration, blood loss, fluid shifts and leak of vitamin D-binding proteins into the interstitial space, may also have contributed to the decrease in circulating 25(OH)D levels in the early postoperative period^(^^2^^,^^21^^)^. Additional supplementation of vitamin D_3_ in these patients may be required.

While the results of the current study are intriguing, the study is limited by its relatively small sample size, precluding the analysis of more definitive endpoints such as mortality or major complications of the surgical procedure. The sample size is also too limited to reliably examine non-linear relationships between the plasma levels of total 25(OH)D, 25(OH)D_2_ and 25(OH)D_3_, and the outcome of interest. However, all patients had plasma levels of total 25(OH)D below 100 nmol/l which is believed to reflect scarcity of 1,25(OH)_2_D, leading to deficient rather than excessive vitamin D effects^(^^1^^)^. Furthermore, the association between PAOF and total 25(OH)D and 25(OH)D_3_ observed in the univariate analysis may have been rendered insignificant in the multivariate model due to lack of power. Thus, it is important to examine this association in a larger study. The blood samples from the patients were collected throughout the year and circulating total 25(OH)D levels were found to be independent of season (data not shown). This is in accordance with work demonstrating that there is no seasonal variation in circulating total 25(OH)D levels in elderly Icelandic patients, who are living in a country which is geographically located at 64°N and where the use of cod liver oil supplementation is common^(^^22^^)^.

The plasma 25(OH)D_2_ levels were lower in our patients than has been reported previously. This finding is hard to explain by analytical measurement uncertainty, the only plausible exception being batch-to-batch variation. However, the samples were run with matched cases and controls placed adjacent to each other in order to avoid this phenomenon. Therefore, batch-to-batch variation is an unlikely explanation for our findings which can most probably be explained by dietary differences. Finally, this association study cannot establish a cause-and-effect relationship as unknown confounding factors may have influenced the results.

In conclusion, higher plasma levels of 25(OH)D_2_ were associated with higher incidence of POAF, whereas no association was found for plasma levels of total 25(OH)D and 25(OH)D_3_. This association of 25(OH)D_2_ with the risk of POAF needs to be addressed in future studies.
